# Multifactorial late complications 13 years after conversion to Roux-en-Y gastric bypass: a case report

**DOI:** 10.1093/jscr/rjag593

**Published:** 2026-08-21

**Authors:** Maximo Vicente Torres Guaicha, Tabata L Tinoco Ortiz, Glenda Y Herrera Cevallos, Amilcar Herrera Cevallos, Paul M Tovar-Cobos

**Affiliations:** Department of Surgery, Hospital Metropolitano de Quito, Avenida Mariana de Jesús S/N y Nicolás Arteta, 170521, Quito, Ecuador; Department of Surgery, Hospital Metropolitano de Quito, Avenida Mariana de Jesús S/N y Nicolás Arteta, 170521, Quito, Ecuador; Department of Surgery, Hospital Metropolitano de Quito, Avenida Mariana de Jesús S/N y Nicolás Arteta, 170521, Quito, Ecuador; Department of Surgery, Hospital Metropolitano de Quito, Avenida Mariana de Jesús S/N y Nicolás Arteta, 170521, Quito, Ecuador; School of Medicine, Universidad San Francisco de Quito, Avenida Diego de Robles S/N y Vía Interoceánica, 170901, Quito, Ecuador

**Keywords:** revisional bariatric surgery, Roux-en-Y gastric bypass, internal hernia, Petersen’s defect, hiatal hernia, sleeve gastrectomy conversion

## Abstract

Late complications after conversion to Roux-en-Y gastric bypass (RYGB) may present many years after the index procedure and often pose significant diagnostic challenges. We report the case of a 51-year-old woman with persistent postprandial upper abdominal pain and worsening gastroesophageal reflux disease 13 years after conversion from sleeve gastrectomy to RYGB. She had previously undergone cholecystectomy without symptom resolution. Imaging suggested incisional hernia, hiatal hernia and internal hernia. Diagnostic laparoscopy revealed a Petersen’s space internal hernia without ischemia, anomalous crossing of the alimentary and biliopancreatic limbs, a sliding hiatal hernia, dense adhesions, and a small incisional hernia. Comprehensive surgical correction was performed, resulting in complete symptom resolution. This case highlights the multifactorial nature of late complications after revisional bariatric surgery and underscores the importance of maintaining a high index of suspicion for complex anatomical causes of persistent abdominal pain.

## Introduction

Obesity remains a major global health challenge, current rising trends will lead to over half of the adult population living with overweight or obesity by 2050, and no country has succesfully reverted these increasing rates [1]. Bariatric surgery is the most effective long-term treatment for sustained weight loss and metabolic improvement [2].

Among surgical options, Roux-en-Y gastric bypass (RYGB) and sleeve gastrectomy (SG) are the most common procedures. RYGB produces greater long-term weight loss, higher type 2 diabetes remission rates, and better gastroesophageal reflux disease (GERD) control [3]. SG is associated with an increased risk of de novo GERD, lower risk of major complications, and nutritional deficiencies [3]. Regardin reintervention RYGB is associated with lower risk of late reintervention when compared to SG and slightly reduced rates of early reintervention [4, 5]. Both procedures have similar incidence of short term complications and mortality rates [6].

Of note, conversion from SG to RYGB is a common indication from refractory GERD, weight regain or suboptimal weight loss. The conversion procedure presents higher complications than primary RYGB though the overall risk remains low (7.2% in conversion vs 5.0% for primary RYGB) [7]. Most notable complications include anastomotic leak (0.5%), bleeding (2.0%), and reoperation (3.0%) [7, 8]. Long term complications for conversion procedure however remain insufficiently characterized.

We present a case illustrating a combination of internal hernia with and anomalous limb positioning, hiatal hernia, and incisional hernia in a patient with previous SG to RYGB conversion 13 years ago. This report aims to emphasize the complex anatomical alterations and clinical consequences of revisional bariatric surgery, as well as the importance of maintaining a high index of suspicion for late and multifactorial complications in patients presenting with persistent abdominal symptoms years after their index procedure.

## Case report

A 51-year-old woman with a long-standing history of GERD refractory to medical treatment presented with postprandial upper abdominal pain for one year, accompanied by nausea and vomiting. She had previously undergone vertical SG 18 years earlier, which was converted to RYGB 13 years ago. Her chief complaint of abdominal pain was initially diagnosed as cholecystitis and she underwent surgical resolution with cholecystectomy 9 months before presentation, but symptoms persisted despite this treatment and GERD had worsened.

On physical examination clinical underweight [body mass index (BMI): 18 kg/m^2^] was noted, a 1.5 cm hernia defect was palpated over the left flank with a protruding mass of 0.5 cm. CT demonstrated prior cholecystectomy, a small amount of fluid in the right paracolic gutter, sliding hiatal hernia and modified gastric anatomy due to previous RYGB procedure with signs suggesting small internal hernia. Upper gastrointestinal endoscopy confirmed a 3 cm sliding hiatal hernia with a hill grade III cardia. Surgical resolution was decided. Diagnostic laparoscopy revealed adhesions (Zuhlke grade IV) attaching the epiploon and small bowel to the abdominal wall on mesogastria and epigastria, as well as interloop adhesions. An abnormal crossing between the alimentary and biliopancreatic limbs was observed, with the alimentary limb passing posterior to the latter. An internal hernia was identified at the Petersen space with the common limb passing through without vascular compromise. The hiatal hernia seen on image studies was localized exhibiting migration of the gastroesophageal junction 3 cm above the hiatus. A left incisional hernia of 1.5 cm was noted with 5 mm fat content.

Surgical correction was performed with adhesiolysis, hiatal repair, revision of previous gastric procedure with reconstruction of gastroenteric anastomosis, closure of Petersen’s mesenteric defect and incisional hernia repair. Postoperatively the patient had an uneventful recovery, she was discharged the following day with oral analgesics and prophylactic PPIs for one week. On subsequent control visits it was noted that the previously low BMI returned to optimal values; routine lab works were under normal ranges, and most importantly; the patient reported complete relief from the postprandial pain and improvement of GERD symptoms.

## Discussion

Late complications after RYGB may appear many years after surgery with upper abdominal pain being the most common chief complaint [9]. In patients who have undergone conversion from SG altered anatomy and adhesions increase diagnostic complexity, as illustrated in this case.

Most common late complications reported in the literature include: anastomotic stricture, bowel obstruction, marginal ulceration, cholelithiasis, incisional hernia, nutritional and vitamin deficiencies, dumping syndrome, malabsorption, gastrogastric fistula and internal hernia [2, 10]. Some of which were present on this patient: Incisional hernia is a potential complication of any surgical procedure, though usually it is more present on open approaches, but it is still reported on laparoscopic bariatric surgery ranging 1%–4% of cases [2].

In contrast, internal hernia is a unique complication of RYGB. It arises from mesenteric defects, classically the Petersen’s space, as seen in this patient. The most important risk of internal hernia is the possibility of bowel obstruction in the herniated defect, although not seen on this case the incidence of this sequence as a late complication was reported to be ~9% in one cohort study [10]. The abnormal crossing of biliopancreatic and alimentary limbs observed in the patient was likely due to the traction of the internal hernia. The position of the alimentary limb suggest a retrocolic approach was performed in the initial SG to RYGB conversion. Nowadays an anterocolic approach is preferred precisely because it lowers the incidence of internal hernia [11].

Cholecystitis was a also a suspected diagnosis in this patient before presentation. Although the rationale for this suspicion wasn’t avaiable to us it doesn’t seem unreasoable to think that the upper abdominal postprandial pain in a patient with RYGB (given the association with cholelitiasis as a late complication) lead in this direction. However, the continuity of symptoms despite surgical resection of the gallblader pointed to another cause.

That cause most likely was either the already discussed internal hernia or the sliding hiatal hernia, a combination of both factors is also plausible. The latter complication would also explain the persistance of GERD symptoms and has been described both as pre-existing condition and as a post-operative outcome [12, 13]. In this case it was most likely a post-operative result since a pre-existence would have been corrected in previous procedures, of note, an exception to this would be type I hiatal hernias that do not necessarily require concomitant repair [14]. For the type III hiatal hernia described on the case an improvement of symptoms are expected following repair [15].

The coexistence of multiple anatomical defects in this patients highlights how persistent abdominal pain after revisional bariatric surgery may be multifactorial rather than attributable to a single cause. This case also remarks how comprehensive intraoperative exploration can allow both identification and correction of all contributing factors in a single procedure, resulting in complete symptom resolution.
